# Highlighting of a LAGLIDADG and a Zing Finger Motifs Located in the pUL56 Sequence Crucial for HCMV Replication

**DOI:** 10.3390/v11121093

**Published:** 2019-11-26

**Authors:** Gaëtan Ligat, Anthony Couvreux, Raphaël Cazal, Sophie Alain, Sébastien Hantz

**Affiliations:** 1 UMR 1092, INSERM, CHU Limoges, Université Limoges, F-87000 Limoges, France; 2 Laboratoire de Bactériologie-Virologie-Hygiène, National Reference Center for Herpesviruses (NRCHV), CHU Limoges, F-87000 Limoges, France

**Keywords:** human cytomegalovirus, terminase, targets, functional domains

## Abstract

The human cytomegalovirus (HCMV) terminase complex is part of DNA-packaging machinery that delivers a unit-length genome into a procapsid. Sequence comparison of herpesvirus homologs allowed us to identify a potential LATLNDIERFL and zinc finger pattern in N-terminal part of pUL56. Recombinant viruses were generated with specific serine or alanine substitutions in these putative patterns. We identified a LATLNDIERFL pattern characteristic of LAGLIDADG homing endonucleases and a metal-binding pattern involving the cysteine and histidine residues C_191_-X2-C_194_-X22-C_217_-X-H_219_ (CCCH) close to the region conferring letermovir resistance. These patterns are crucial for viral replication, suggesting that they are essential for pUL56 structure and function. Thus, these patterns represent potential targets for the development of new antivirals such as small molecules or peptides and may allow to better understand the letermovir mechanism of action.

## 1. Introduction

The human cytomegalovirus (HCMV) is a betaherpesvirus responsible for significant morbidity and mortality in immunocompromised patients, especially among transplant recipients and in congenital infection. To date, current antiviral inhibitors including ganciclovir, cidofovir and foscarnet target the viral DNA polymerase. One of the limitations of these antivirals is their significant dose-limiting toxicity. Furthermore, they have a poor oral bioavailability and prolonged treatment can cause cross-resistance [1,2,3,4]. Thus, developing new drugs not targeting the viral polymerase, pUL54, is essential. A promising therapeutic alternative, the quinazoline letermovir interferes with the HCMV DNA-packaging stage. In phase II trials, letermovir was effective in prevention of HCMV infection in allogenic hematopoietic-cell recipients with an excellent safety profile [5], data recently confirmed in a Phase III trial (clinicaltrials.gov NCT02137772). Mechanistic studies and the localization of resistance mutations suggested that this drug essentially interacts with the pUL56 subunit of the viral terminase complex (pUL89-pUL56) [6,7,8]. However, resistance observed both in pUL56 and pUL89 and proximity of the selected *UL89* and *UL56* mutations suggests that letermovir targets a functional locus involving pUL56 and pUL89 interaction [9]. The lack of structural and functional data must be filled to better understand the letermovir mechanism and design additional drugs or peptides.

The HCMV terminase complex (pUL89-pUL56) is a critical component of the DNA-packaging machinery which aims to translocate a unit of viral DNA genome into an empty capsid [9]. These proteins are partially characterized through their homology with HSV-1 pUL15 and pUL28, which have an essential role in HSV-1 genome packaging. An interaction between HSV-1 pUL15 and the C-terminal part of pUL28 was demonstrated by coimmunoprecipitation experiments using proteins from HSV-1-infected cells [10]. Genetic experiments have identified several critical domains within the individual HSV-1 terminase subunits that are essential for functioning [11,12]. Regarding HCMV subunits, although pUL89 has shown activities crucial for DNA-packaging process [13], evidence suggests that the large subunit pUL56 has an essential role in this process, carrying many of the functional domains required for DNA-packaging.

pUL56 is the large subunit of HCMV terminase complex and is the gene product of ORF UL56 located on the unique long portion of the viral genome. Its sequence is composed of 12 conserved regions annotated from I to XII [14]. This highly conserved protein of about 130 kDa can establish interactions with many partners. First, it has been observed by cryo-microscopy that pUL56 tends to self-assemble, leading to a functional homodimer [15]. It has also been proven that it interacts with importin alpha through a Nuclear Localization Signal pattern located in its very C-terminal (amino acids 816–827) for its subsequent translocation into the nucleus, to achieve maturation and translocation of viral DNA with the help of its terminase complex partners. The translocation process is partly powered by its ATPase activity, which is enhanced when it is associated with pUL89 [16] through the short sequence _671_WMVVKYMGFF_680_ [17]. The maturation of DNA consists in its cleavage from linear to single unit length viral DNA. Whereas pUL89 is able to cleave non-specific DNA strands through an RNase H/integrase-like fold, pUL56 is able to recognize, and subsequently cleave, “pac” motifs (“cis-acting packaging signal”) located in the “a” sequence of the terminal and internal repeat segments [18].

In a previous study, we identified, by in silico analysis of pUL56 sequence, a highly conserved region that could form a putative zinc-finger pattern in three different ways C_191_-X2-C_194_-X22-C_217_-X-H_219_ (CCCH), C_191_-X2-C_194_-X22-C_217_-X5-H_223_ (CCCH), and C_191_-X2-C_194_-X24-H_219_-X3-H_223_ (CCHH) [14]. Interestingly, a Q204R mutation that confers BDCRB resistance is located within this region [19].

To date, neither overexpression nor homology modeling succeeded in obtaining a reliable structure of pUL56. To address the question of the pUL56 nuclease mechanism, we proceeded with a meticulous analysis of its primary sequence and predicted secondary structures. This work was combined with the state-of-the-art concerning nuclease structures reviewed by Yang in 2011 [20]. Nucleases fall into more than twelve families, depending on function, structure or substrate. Although the core structure of most of them folds into complicated β and α arrangements (e.g., DnaQ-like, RnaseH, FEN like, REC-J families), simple β-hairpins are also encountered in DEK and Rnase II members. Despite those strong structural discrepancies, one or more carboxylate amino acids are in the catalytic site center to bind divalent ions and a water molecule, or a histidine in some cases, constituting the nucleophilic component of a SN2 reaction for DNA (RNA) cleavage. Homing endonucleases are another family of nucleases widely represented over all branches of life. Among their members, we can distinguish the LAGLIDAGD group. 

In this report, we present sequences analysis which allows to assume the existence of a _134_LATLNDIERFL_144_ pattern characteristic of LAGLIDADG homing endonuclease. This protein family recognizes specific DNA sequences for their subsequent cleavage. The structure of several members of LAGLIDADG homing endonucleases has already been resolved at the atomic level [21,22,23]. Interestingly, some homodimeric members of that family have been widely studied. I-CreI (PDB: 1G9Y) and I-CeuI (PDB: 2EX5) structures present a highly specific two-fold symmetry axis, located in the very N-terminus of each subunit of the homodimer. LAGLIDADG patterns constitute one side of a leucine-zipper through conserved hydrophobic amino acids (i.e., leucine and isoleucine). This tertiary structure allows to constitute a negatively charged cluster of carboxylate residues (i.e., aspartates) able to chelate positively charged ions (mostly calcium or magnesium) essential for DNA cleavage. Moreover, LAGLIDADG homing endonucleases have a DNA recognition region (DRR) motif located one hundred residues downstream from the LAGLIDADG pattern [24]. In this report, we present sequence analysis which suggests the existence of a _134_LATLNDIERFL_144_ pattern characteristic of LAGLIDADG homing endonuclease. We used mutational approaches to investigate putative functions of the LATLNDIERFL and DNA binding patterns of pUL56. We confirm the presence of the LATLNDIERFL pattern characteristic of LAGLIDADG homing endonuclease and a metal-binding motif involving the cysteine and histidine residues within the sequence C_191_-X2-C_194_-X22-C_217_-X-H_219_ (CCCH) near the region conferring letermovir resistance [25]. We propose that these two motifs are essential for pUL56 structure and function and represent potential antiviral targets. Taken together, we propose that the pUL56 subunit of terminase complex belongs to the LAGLIDADG homing endonuclease family.

## 2. Materials and Methods

### 2.1. Identification of Conserved Patterns and Secondary Structure Prediction

The pUL56 amino acid sequence of reference strain AD169 [26] was aligned with the sequences of 21 homologous proteins from other herpesviruses, as described in Table S1 (supplementary data). Alignments were performed with Clustal Omega (Ω) multiple sequence alignment (MSA) tool, provided by the EMBL-EBI bioinformatics web and programmatic tools framework [27,28,29].

### 2.2. Cells and Bacterial Strains

Human fibroblasts MRC-5 (bioMérieux, Craponne, France) were cultivated at 37 °C in 5% CO_2_ and grown in minimal essential medium (MEM) containing 10% fetal bovine serum with antimicrobials. Escherichia coli strain GS1783 was used for BAC mutagenesis [30]. The HCMV-BAC (Bacterial Artificial Chromosome containing the genome of the CMV laboratory strain AD169) contains an enhanced green fluorescent protein (EGFP) gene in the unique short region and was derived from parental strain pHB5, the BAC-cloned genome of the CMV laboratory strain AD169 [26].

### 2.3. BAC Mutagenesis

To identify the crucial amino acids implied in putative LATLNDIERFL and zinc finger patterns, highly conserved residues were substituted with a serine or an alanine by “en passant” mutagenesis, a two-step markerless Red recombination system for BAC mutagenesis in *E. coli* strain GS1783. Single UL56 mutations were introduced into an EGFP-expressing HCMV-BAC [30] to generate several mutants, as described in Table 1. Primers used for mutagenesis are described in Tables S2 and S3. Presence of mutations in *UL56* gene of each virus was confirmed by sequencing prior to transfection. We previously showed that the “en passant” mutagenesis does not introduce other mutations that could have a negative impact on viral replication [16]. The mutant Q204R was tested in the same experiments as positive control [19].

### 2.4. Reconstitution of Viruses Harboring the Mutations

The impact of all mutations on viral growth was assessed using transfection of mutated HCMV-BAC into human fibroblasts MRC-5 (Biomerieux, France), using the liposomal reagent Transfast^TM^ (Promega, Madison, WI, USA), following manufacturer’s instructions.

### 2.5. Plaque Assays and Growth Curve Analysis

To estimate the impact of each mutation on viral fitness, we inoculated viral recombinant strains and AD169-EGFP in 48-well MRC-5 culture with a multiplicity of infection (MOI) of 0.01. From day 1 to day 7 post-inoculation, the number of fluorescent cytopathic foci was counted to establish viral growth curves for each recombinant. Curves represent the average of three independent experiments. 

### 2.6. Viral Immediate Early and Late Proteins Expression

A transfection of mutated HCMV-BAC into human fibroblasts MRC-5 using the liposomal reagent Transfast^TM^ (Promega, USA) was performed. Cells were fixed at 5 days post transfection, and immunostaining was performed for viral immediate early (anti-IE1 antibody; Argene, France) and late (anti-gB antibody; Abcam, UK) proteins in transfected cells.

## 3. Results

### 3.1. Identification of a LATLNDIERFL Pattern into pUL56, Characteristic of the LAGLIDADG Homing Endonuclease

**Figure 1:**
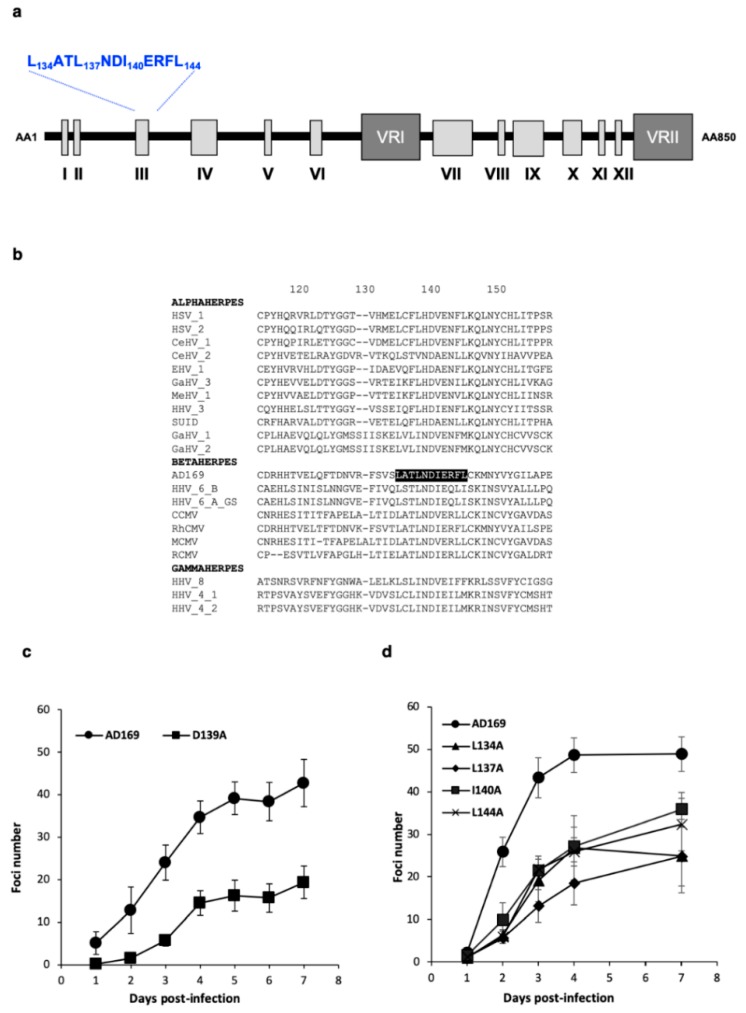
Identification of a putative LATLNDIERFL pattern in N-terminal part of pUL56. (**a**) Structure of HCMV pUL56 (according to [14]) with a putative LATLNDIERFL pattern (blue word). (**b**) Sequences alignment of conserved regions from 21 herpesviruses belonging to α, β and γ sub-families of herpesviruses. Sequence numbering is consistent with that of the HCMV reference strain AD169 residues. Key residues involved in the formation of the putative LATLNDIERFL of pUL56 pattern are shown as white letters on a black background. (**c**) Growth curves of the recombinant virus strains HCMV-BAC *UL56* D139A in comparison to the parental strain HCMV-BAC AD169. Fluorescent foci were counted daily from day 1 to day 7. (**d**) Growth curves of the recombinant virus strains HCMV-BAC *UL56* L134A, L137A, I140A and L144A in comparison to the parental strain HCMV-BAC AD169. Fluorescent foci were counted daily from day 1 to day 7. Curves are the average of three independent experiments.

Comparing the protein sequence of 21 homologous proteins of pUL56 from other herpesviruses, we identified an amino acid sequence characteristic of the LAGLIDADG pattern of the LAGLIDADG homing endonuclease family, located in the N-terminal part of pUL56 (Figure 1a,b and Figure S1). This region of the pUL56 protein is a well-conserved sequence, particularly regarding the amino acids physico-chemical characteristics. However, even within LAGLIDADG homing endonucleases, this characteristic pattern is not highly conserved, and only the second aspartic acid is conserved among LAGLIDADG homing endonuclease [31].

### 3.2. Several Amino Acids of pUL56 Putative _134_LATLNDIERFL_144_ Pattern are Essential for Viral Replication

To identify amino acids of _134_LATLNDIERFL_144_ pattern potentially involved in dimerization and/or endonuclease activity we produced by “en passant” mutagenesis several recombinant EGFP-viruses. 

First, we mutated two conserved negatively charged amino acids (D139, E141), predicted to be involved in the chelation of positively charged ions (mostly calcium or magnesium), essential for DNA cleavage. Contrary to the substitution of D139A, the E141A mutation completely impaired viral replication (Table 1 and Figure S2). However, the growth curves of the wild-type HCMV-BAC and D139A mutated strains were different, showing that D139A mutation had an impact on HCMV replication capacity (Figure 1c). Indeed, this mutation reduces the capacity of the virus to produce infectious particles. 

Leucine-zipper through conserved hydrophobic amino acid (leucine and isoleucine) are likely to be involved in protein dimerization, as shown for LAGLIDADG homing endonuclease. We assumed that the replacement of these amino acids by alanine would probably prevent the putative dimerization of pUL56. Indeed, mutations of single leucine or isoleucine residues (L134, L137, I140, L144) forming the hypothetical leucine-zipper pattern reduced both the capacity of the virus to produce infectious particles (Table 1) and the viral fitness (Figure 1d). Combinations of only two leucine and isoleucine mutations in this pattern completely impaired viral replication and propagation in cell-culture (Table 1 and Figure S3). 

**Figure 2:**
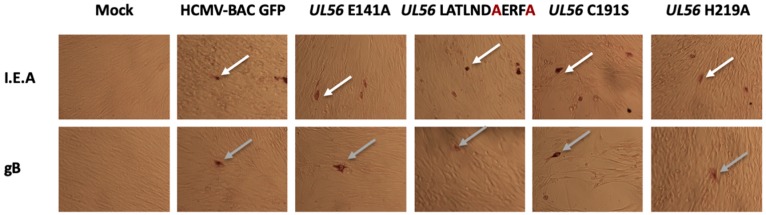
pUL56 are not required for viral late gene expression. MRC-5 were transfected with HCMV-BAC WT or the mutants. Five days after transfection, immunostaining was performed for early (I.E.A) (white arrow) and late (gB) (grey arrow) viral proteins. (Magnification: 100×).

To check if these amino acids substitutions may disrupt another step of the HCMV replication, immunostaining assays were performed to detect proteins produced at immediate early and late stages of viral cycle (IEA and late proteins). Expression of immediate early (IEA) and late (gB) viral genes were detected, indicating that substitutions have no impact on viral gene expression (Figure 2).

As shown previously [16], NGS sequencing on both the original BAC and the mutants, ensuring that no other mutations that could have a negative impact on viral replication, was introduced in the BAC backbone during the manipulations. Mutations were found in 100% of the mutant BAC sequences, whereas other SNPs were located in genes non-essential for viral replication and represent less than 30% of the sequences both in the original BAC and in the mutants.

### 3.3. The Putative Zinc Finger Pattern of pUL56 is Required for Viral Replication

**Figure 3:**
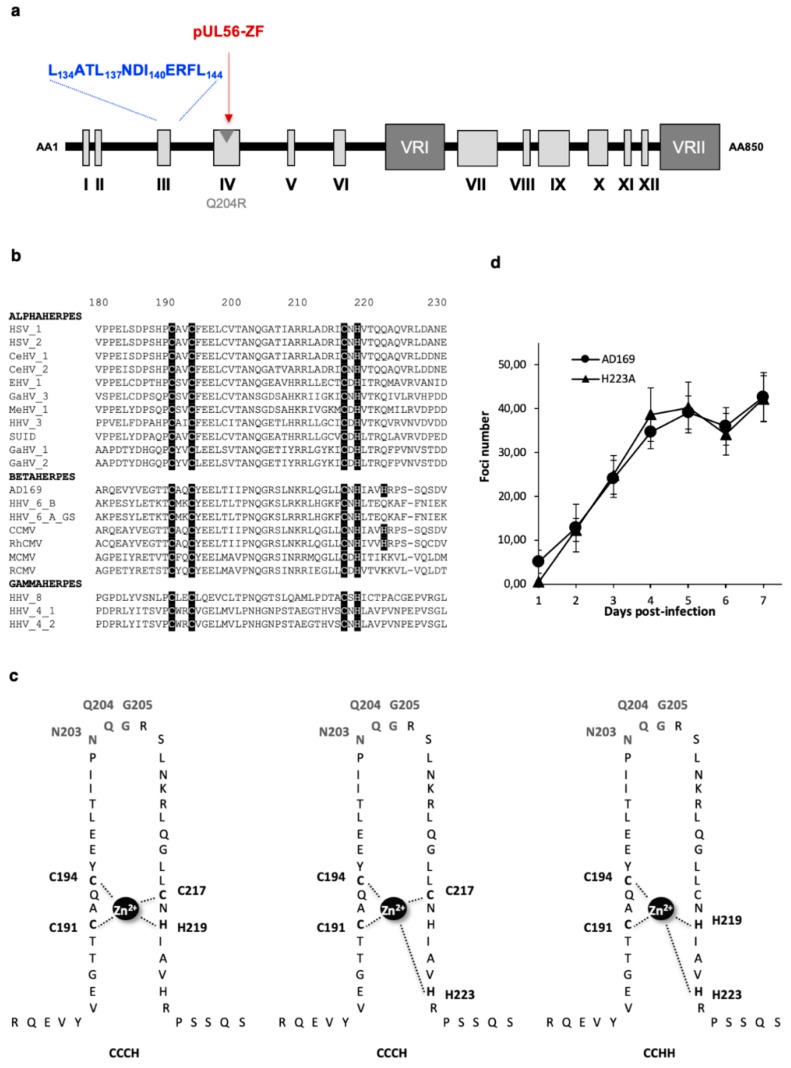
Conserved zinc finger region and amino acids in terminase pUL56. (**a**) Structure of human cytomegalovirus (HCMV) pUL56 (according to [14]) with a putative zinc-finger (ZF) pattern (red word). The position of the Q204R benzimidazole resistance mutation is shown by a grey arrow. (**b**) Sequences alignment of conserved regions IV from 21 herpesviruses and residues involved in metal-binding site [14]. Sequence numbering is consistent with that of the HCMV reference strain AD169 residues. Key residues involved in the formation of the zinc-finger motif are shown as white letters on a black background. (**c**) Three different representations of the putative zinc finger motif: CX_2_CX_22_CXH (CCCH zinc finger), CX_2_CX_22_CX_5_H (CCCH zinc finger), and CX_2_CX_24_HX_3_H (CCHH zinc finger). Amino acids implicated in the motif are highlighted: (C191, C194, C217 and H219), (C191, C194, C217 and H223) and (C191, C194, C219 and H223) respectively. (**d**) Growth curves of the recombinant virus strains HCMV-BAC UL56 H223A in comparison to the parental strain HCMV-BAC AD169. Fluorescent foci were counted daily from day 1 to day 7. Curves are the average of three independent experiments.

The pUL56 subunit of the terminase complex displays a putative DNA binding pattern with a presumed zinc finger motif (Figure 3a,b and Figure S1) [18]. To investigate the putative DNA binding pattern of pUL56, previously described in three different ways—C_191_-X2-C_194_-X22-C_217_-X-H_219_ (CCCH), C_191_-X2-C_194_-X22-C_217_-X5-H_223_ (CCCH), and C_191_-X2-C_194_-X24-H_219_-X3-H_223_ (CCHH) (Figure 3c) [18]—we produced by “en passant” mutagenesis eight recombinant EGFP-viruses (C191S, C194S, C217S, H219A, H223A, N203A, Q204R, G205A) (Table 2 and Figure S4). 

Cysteines 191, 194 and 217, and histidine 219 and 223, were selected for mutagenesis because these residues were predicted to be involved in the chelation of the zinc ion essential for zinc-finger structuration. Residues N203 and G205 were selected for mutagenesis because they are perfectly conserved among all the 20 herpesvirus homologues of pUL56 and flank the previously described Q204R resistance mutation. The Q204R mutant was tested in the same experiments as positive control.

Eleven days after the transfection of human fibroblasts, we observed no cytopathic effect for the subsequent mutations C191S, C194S, C217S, H219A and N203A, located within the putative metal-binding motif (Table 2). We found that most missense mutations dramatically impaired viral replication and virions production. In contrast, two mutations H223A and G205A did not alter viral replication (Table 2 and Figure S4). Growth curves of the wild-type HCMV-BAC and H223A mutated strains were similar, showing that the H223 mutation had no impact on HCMV replication capacity (Figure 3d).

As for the LATLNDIERFL pattern experiments, expression of immediate early (IEA) and late (gB) viral genes were detected, indicating that the tested substitutions have no impact on viral gene expression (Figure 2).

## 4. Discussion

Nucleosides analogues such as ganciclovir or cidofovir target the viral DNA polymerase of HCMV. There are a few problems associated with these treatments, such as the emergence of resistance in immunosuppressed individuals and toxicity. Thus, there is a clinical need to develop new therapies to treat HCMV infection, and identifying novel targets could be an interesting option.

The process of herpesvirus DNA packaging requires the involvement of terminases. The functional packaging holocomplex is a hetero-oligomer composed of pUL56, pUL89 and pUL51 proteins. HCMV packaging initiates when a packaging signal called pac sequence is recognized on concatemeric DNA by the viral terminase complex. The DNA/terminase complex then binds to an empty procapsid at its unique portal vertex, embedded into the preformed procapsid. DNA is then translocated through the portal vertex. A second site-specific cleavage occurs to terminate packaging when a unit length genome has been translocated.

Sequence alignment showed a strong homology between pUL56 and LAGLIDADG homing endonucleases with a zing-finger and a _134_LATLNDIERFL_144_ patterns located in pUL56 N-terminal region. As pUL56, LAGLIDADG Homing endonucleases recognize DNA molecules and are involved in DNA nuclease activity [21,24,32].

To investigate the putative _134_LATLNDIERFL_144_ and zing finger patterns of pUL56, we produced by “en passant” mutagenesis recombinant EGFP-viruses. We found several residues crucial for viral replication within the LATLNDIERFL and the zinc-finger patterns of pUL56. Interestingly, although our recombinant L134A reduced the viral fitness, the variant L134V was sensitive to letermovir, with no impact on viral fitness, thus representing a natural polymorphism [33]. Both aliphatic amino acids leucine and valine have a longer carbon chain than alanine. Therefore, L134A mutation may alter the dimerization of pUL56 through the _134_LATLNDIERFL_144_ pattern, and so decrease the efficacy of replication, whereas this dimerization could still be possible with the L134V mutant. 

**Figure 4:**
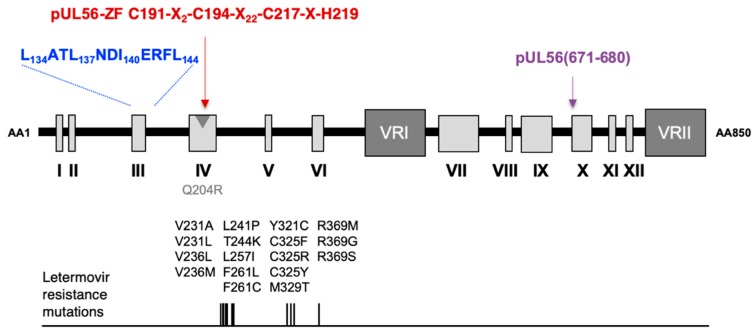
Proposed model of structure of HCMV terminase subunit pUL56 with the LATLNDIERFL (blue word) and the zinc finger within the sequence C191-X_2_-C194-X_22_-C217-X-H219 (red word), according to [18]. The LATLNDIERFL and the zinc finger patterns are located near the pUL56 region that includes mutations that facilitate letermovir resistance. pUL56 (671-680) (purple word), shown previously as a binding site for pUL89 [16]. The position of the Q204R benzimidazole resistance mutation is shown by a grey arrow.

As found in LAGLIDADG homing endonucleases, we propose that two subunits of pUL56 use the _134_LATLNDIERFL_144_ pattern as an interface domain where hydrophobic amino acids L134, L137, I140 and L144 dimerize. Acidic residue E141 may contribute to the active sites, where they participate in positively charged ions chelation (mostly calcium or magnesium) essential for DNA cleavage. Residues C191, C194, C217 and H219 that are part of a cysteine-rich metal-binding motif, are essential for viral replication. We propose that a metal-binding motif involving the cysteine and histidine residues within the sequence C_191_-X2-C_194_-X22-C_217_-X-H_219_ is essential for pUL56 function. Side chain of polar amino acid N203 may recognize DNA through hydrogen bonds interactions. These results suggest that the cysteine-rich metal-binding motif, in the N-terminal of pUL56, may be required for both pUL56 structure and function, either for DNA-binding and/or nuclease activity. Thus, the association of two _134_LATLNDIERFL_144_ patterns and two zinc-fingers could constitute the pUL56 DNA-binding and cleavage site (Figure 4). 

Interestingly, the _134_LATLNDIERFL_144_ and zinc-finger patterns are located near the pUL56 region encompassing mutations that facilitate letermovir resistance (Figure 4). Thus, these motifs could be involved in the mechanism of action of letermovir.

In this study, our data confirmed an essential role of pUL56 during HCMV infection and showed that both the LATLNDIERFL and the zinc-finger patterns in the N-terminal of sequence of pUL56 are crucial for viral replication. We hypothesize that the pUL56 subunit of the terminase complex belongs to the LAGLIDADG homing endonucleases. These findings suggest that these patterns are a prime target for the development of new antiviral such as small molecules, peptides that could interfere with pUL56 functions, and could maybe allow a better understanding of the letermovir mechanism of action. Although mutational approaches are often employed to investigate putative functional domains into proteins [17,34], functional studies are required to confirm our hypothesizes. Thus, future studies will examine if these motifs are involved in the nuclease activity of pUL56.

## Figures and Tables

**Table 1:** Overview of HCMV-BAC-*UL56* mutants in putative LATLNDIERFL pattern with their respective amino acid exchanges and the mutation effect on viral replication (foci).

Amino Acids ^a^	Mutation(s)	Mutant HCMV-BAC	Day 4 Post-Tranfection	Day 11 Post-Transfection
*UL56* WT	/	/	-	++
LATLN**D**IERFL	LATLN**A**IERFL	*UL56* D139A	-	+
LATLNDI**E**RFL	LATLNDI**A**RFL	*UL56* E141A	-	-
**L**ATLNDIERFL	**A**ATLNDIERFL	*UL56* L134A	-	+
LAT**L**NDIERFL	LAT**A**NDIERFL	*UL56* L137A	-	+
LATLND**I**ERFL	LATLND**A**ERFL	*UL56* I140A	-	+
LATLNDIERF**L**	LATLNDIERF**A**	*UL56* L144A	-	+
**L**AT**L**ND**I**ERF**L**	**A**AT**A**ND**A**ERF**A**	*UL56* L134A L137A I140A L144A	-	-
LATLND**I**ERF**L**	LATLND**A**ERF**A**	*UL56* I140A L144A	-	-
**L**ATLNDIERF**L**	**A**ATLNDIERF**A**	*UL56* L134A L144A	-	-

^a^ Amino acids changed in the mutants are in bold. /: no change; -: absence of viral growth; +: small foci; ++: foci; HCMV-BAC: Bacterial Artificial Chromosome containing the genome of the CMV laboratory strain AD169.

**Table 2:** Overview of HCMV-BAC-*UL56* mutants in putative zinc-finger pattern with their respective amino acid exchanges and the mutations’ effect on viral replication (foci).

Amino Acids ^a^	Mutation(s)	Mutant HCMV-BAC	Day 4 Post-Transfection	Day 11 Post-Transfection
*UL56* WT	/	/	-	++
**C**-C-NQG-C-H-H	**S**-C-NQG-C-H-H	*UL56* C191S	-	-
C-**C**-NQG-C-H-H	C-**S**-NQG-C-H-H	*UL56* C194S	-	-
C-C-NQG-**C**-H-H	C-C-NQG-**S**-H-H	*UL56* C217S	-	-
C-C-NQG-C-**H**-H	C-C-NQG-C-**A**-H	*UL56* H219A	-	-
C-C-NQG-C-H-**H**	C-C-NQG-C-H-**A**	*UL56* H223A	-	++
C-C-**N**QG-C-H-H	C-C-**A**QG-C-H-H	*UL56* N203A	-	-
C-C-N**Q**G-C-H-H	C-C-N**R**G-C-H-H	*UL56* Q204R	-	+
C-C-NQ**G**-C-H-H	C-C-NQ**A**-C-H-H	*UL56* G205A	-	+

^a^ Amino acids changed in the mutants are in bold. /: no change; -: absence of viral growth; +: small foci; ++: foci; HCMV-BAC: Bacterial Artificial Chromosome containing the genome of the CMV laboratory strain AD169.

## Supplementary Materials

The following are available online at https://www.mdpi.com/1999-4915/11/12/1093/s1, Figure S1: Identification of patterns in N-terminal part of pUL56 from four reference strains (AD169, TB40/E, Toledo, Towne), Figure S2: Effect of the terminase pUL56 D139A and E141A mutations on viral growth, Figure S3: Effect of the terminase pUL56 mutations within the putative leucine-zipper on viral growth, Figure S4: Effect of the terminase pUL56 mutations within the putative zinc finger on viral growth, Table S1: Sequences used for alignment of pUL56 and homologues, Table S2: Sequences of UL56-primers used for mutagenesis-PCR into putative LATLNDIERFL pattern, Table S3: Sequences of UL56-primers used for mutagenesis-PCR into putative zinc-finger pattern.
