# Supplementary material for: Highlighting of a LAGLIDADG and a Zing Finger Motifs Located in the pUL56 Sequence Crucial for HCMV Replication
Source: Viruses. 2019 Nov 26;11(12):1093. doi: 10.3390/v11121093 (PMC6950143; doi:10.3390/v11121093)
Supplement: Supplementary file 1 [file viruses-11-01093-s001.pdf]

Supplementary Materials

Highlighting of a LAGLIDADG and a zing finger motifs located in the pUL56 sequence crucial for HCMV replication

Gaëtan Ligat[1,2*#], Anthony Couvreux[1], Raphaël Cazal[1], Sophie Alain[1,2], Sébastien Hantz[1,2*]
*correspondence to: gligat@unistra.fr, sebastien.hantz@unilim.fr

a

```
AD169    CDRHHTVELQFTDNVRFSVSLATLNDIERFLCKMNYVYGILAPE
TB40/E   CDRHHTVELQFTDNVRFSVSLATLNDIERFLCKMNYVYGILAPE
Toledo   CDRHHTVELQFTDNVRFSVSLATLNDIERFLCKMNYVYGILAPE
Towne    CDRHHTVELQFTDNVRFSVSLATLNDIERFLCKMNYVYGILAPE
```

b

```
AD169    ARQEVYVEGTTCAQCYEELTIIPNQGRSLNKRLQGLLCNHIAVHRPSSQSDV
TB40/E   ARQEVYVEGTTCAQCYEELTIIPNQGRSLNKRLQGLLCNHIAVHRPSSQSDV
Toledo   ARQEVYVEGTTCAQCYEELTIIPNQGRSLNKRLQGLLCNHIAVHRPSSQSDV
Towne    ARQEVYVEGTTCAQCYEELTIIPNQGRSLNKRLQGLLCNHIAVHRPSSQSDV
```

Figure S1: Identification of patterns in N-terminal part of pUL56 from 4 reference strains (AD169, TB40/E, Toledo, Towne). a. Sequences alignment of the LATLNDIERFL pattern. b. Sequences alignment of conserved regions IV and residues involved in metal-binding site. Key residues involved in the both patterns are shown as white letters on a black background.

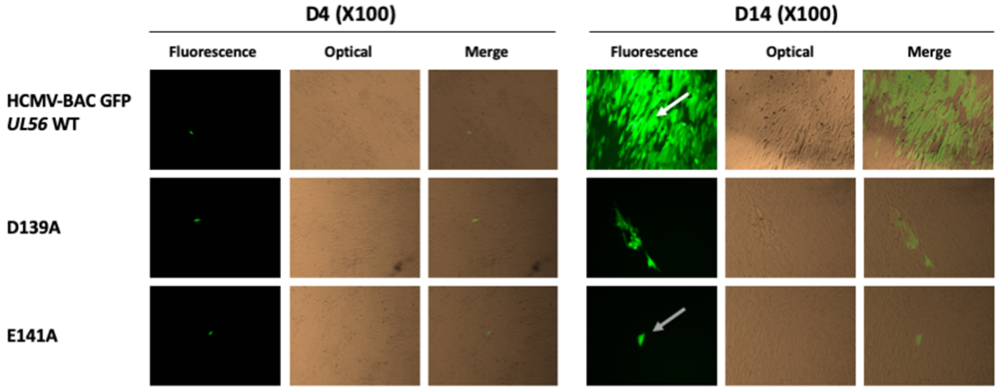

Figure S2: Effect of the terminase pUL56 D139A and E141A mutations on viral growth. A. Plaque formation assay using tranfection of HCMV-BAC AD169 and recombinant virus strains (amino acid probably involved in nuclease activity) in MRC-5 cells at day 4 and day 14. Green fluorescent foci (white arrow) were observed with the wild-type HCMV-BAC GFP and the D139A mutation. Single infected cells (grey arrow) were observed with the E141A mutation.

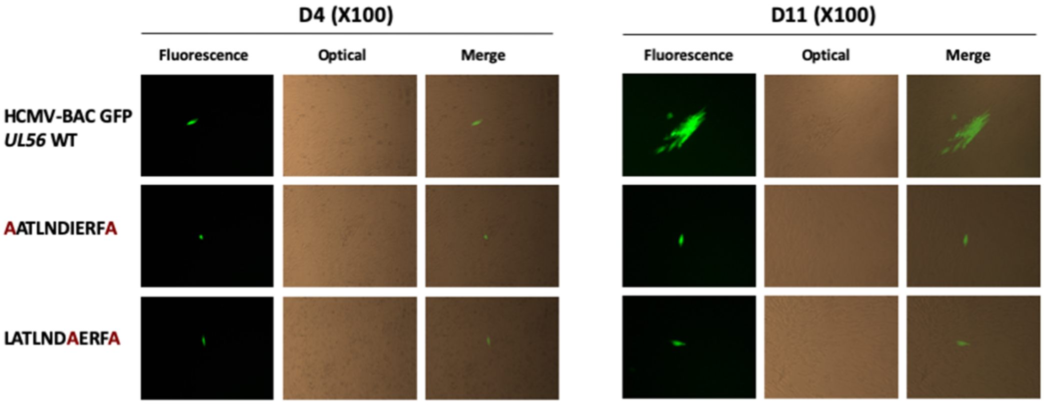

Figure S3: Effect of the terminase pUL56 mutations whithin the putative leucine-zipper on viral growth. Plaque formation assay using tranfection of HCMV-BAC AD169 and recombinants virus strain (amino acid probably involved in leucine zipper pattern) in MRC-5 cells.

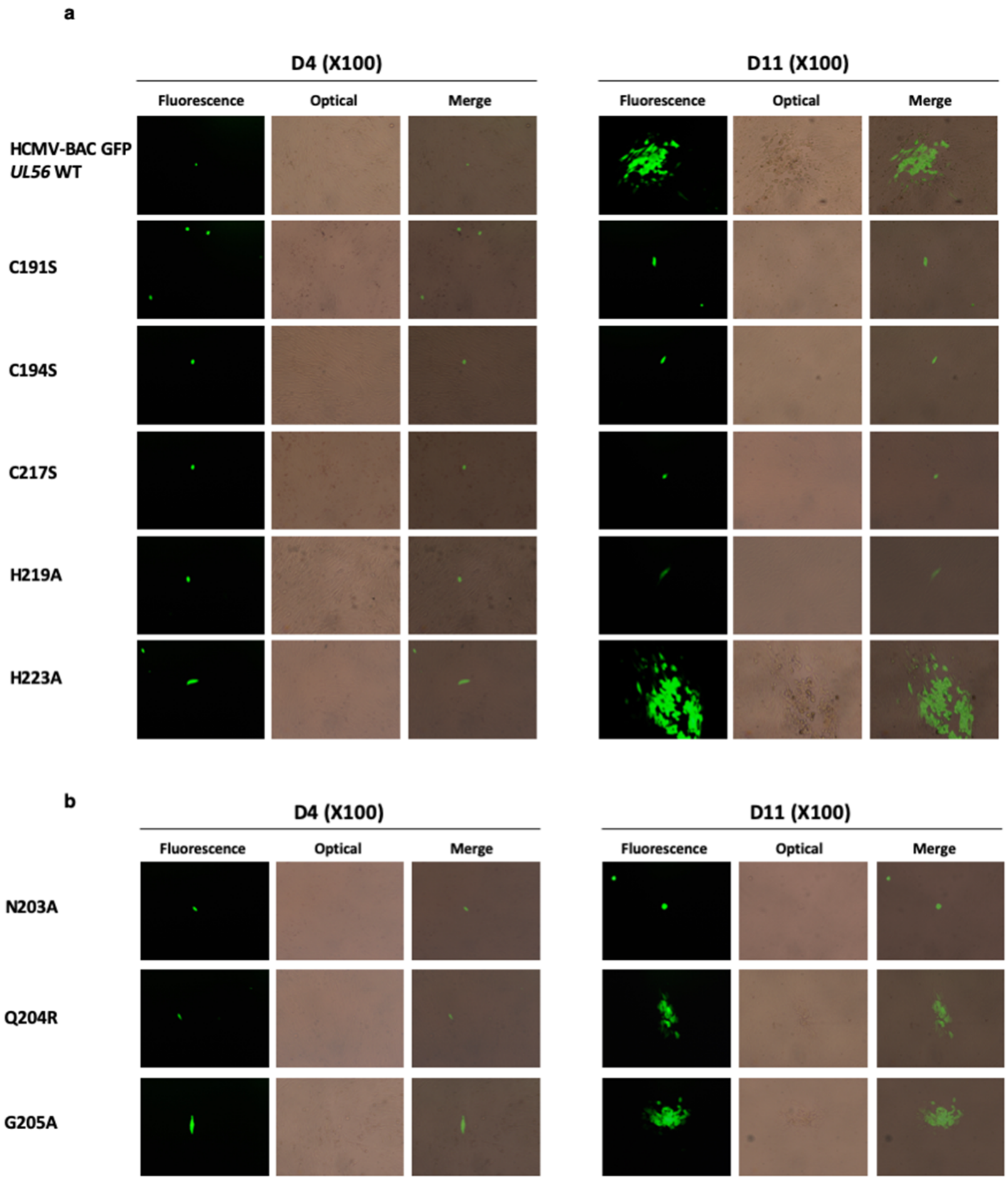

Figure S4: Effect of the terminase pUL56 mutations within the putative zinc finger on viral growth. A. Plaque formation assay using transfection of HCMV-BAC AD169 and recombinants virus strain (amino acid probably involved in zinc chelation of putative zinc finger pattern) in MRC-5 cells. B. Plaque formation assay using transfection of HCMV BAC AD169 and recombinant virus strains (amino acids in the loop of putative zinc finger pattern) in MRC-5 cells.

Table S1: Sequences used for alignment of pUL56 and homologues.

Identification	Sequence identification	Accession number	Reference
HSV_1	Human herpesvirus type 1 pUL28 packaging protein	CAA32321	Dolan et al., 1992
HSV_2	Human herpesvirus type 2 pUL28 packaging protein	CAB0675	Barnett et al., 1992
CeHV_1	Cercopithecine herpesvirus 1 pUL28	BAC58068	Pelyrigina et al., 2003
CeHV_2	Cercopithecine herpesvirus 2 pUL28	AAU84532	Tyler, Peters, and Severini, 2005
EHV_1	Equine herpesvirus 1 ORF32	YP_053077	Telford et al., 1992
GaHV_3	Gallid herpesvirus 3 pUL28	BAB16538	Izumiya et al., 2001
MeHV_1	Meleagrid herpesvirus 1 pUL28 processing and transport protein	AAG30068	Kingham et al., 2001
HHV_3	Human herpesvirus 3 (Varicella-Zooster virus)	CAA27913	Davison and Scott, 1986
SUID	Suid herpesvirus 1 (Pseudorabies virus) UL28	YP_068331	Klupp et al., 2004
GaHV_1	Gallid herpesvirus 1 UL28-like protein	YP_182357	Thureen et al., 2006
GaHV_2	Gallid herpesvirus 2 Homologue of HSV 1 ICP 18.5 packaging protein	AAF66763	Kato et al., 1999
AD169	Human cytomegalovirus AD169 strain pUL56	X17403	Chee et al., 1990
HHV_6_B	Human herpesvirus 6 type B transport protein	Q9WT24	Isegawa et al., 1999
HHV_6_A_GS	Human herpesvirus 3 type A transport protein	AHK06969	None
CCMV	Chimpanzee herpesvirus pUL56	AAM00906	Davison et al., 2003
RhCMV	Cercopithecine herpesvirus 8 (Rhesus cytomegalovirus) putative pUL56 DNA packaging protein	AAZ80592	None
MCMV	Murid herpesvirus 1 (Murine cytomegalovirus) major DNA-binding protein	CAA47415	Messerle et al., 1992
RCMV	Murid herpesvirus 2 (Rat cytomegalovirus Maastricht) pR56 protein	AAC56431	Beuken et al., 1996
HHV_8	Human herpesvirus 8 BALF3 EBV homolog, transport protein homologue	AAC57084	Moore et al., 1996
HHV_4_1	Human herpesvirus 4 type 1 BALF3	YP_401715	de Jesus et al., 2003
HHV_4_2	Human herpesvirus 4 type 2 BALF3	YP001129509	Dolan A et al., 2006

Table S2: Sequences of *UL56*-primers used for mutagenesis-PCR into putative LATLNDIERFL pattern.

Mutant BAC	Primer direction	Primer sequence
UL56 D139A	Forward	GGACAACGTCCGTTTCAGCGTCAGTCTGGCCACACTCAACGCCATCGAGCGCTTCCTG TGCAATAGGGATAACAGGGTAATCGATTT
	Reverse	TCCCGTACACGTAGTTCATTTTGCACAGGAAGCGCTCGATGGCGTTGAGTGTGGCCAG ACTGAGCCAGTGTTACAACCAATTAACC
UL56 E141A	Forward	CGTCCGTTTCAGCGTCAGTCTGGCCACACTCAACGACATCGCCCGCTTCCTGTGCAAAA TGAATAGGGATAACAGGGTAATCGATTT
	Reverse	CCAGGATCCCGTACACGTAGTTCATTTTGCACAGGAAGCGGGCGATGTCGTTGAGTGT GGCCAGCCAGTGTTACAACCAATTAACC
UL56 L134A	Forward	GGAGCTCCAGTTTACGGACAACGTCCGTTTCAGCGTCAGTGCGGCCACACTCAACGAC ATCGATAGGGATAACAGGGTAATCGATTT
	Reverse	TCATTTTGCACAGGAAGCGCTCGATGTCGTTGAGTGTGGCCGCACTGACGCTGAAACG GACGTGCCAGTGTTACAACCAATTAACC
UL56 L137A	Forward	GTTTACGGACAACGTCCGTTTCAGCGTCAGTCTGGCCACAGCCAACGACATCGAGCGC TTCCTTAGGGATAACAGGGTAATCGATTT
	Reverse	ACACGTAGTTCATTTTGCACAGGAAGCGCTCGATGTCGTTGGCTGTGGCCAGACTGAC GCTGAGCCAGTGTTACAACCAATTAACC
UL56 I140A	Forward	CAACGTCCGTTTCAGCGTCAGTCTGGCCACACTCAACGACGCCGAGCGCTTCCTGTGC AAAATTAGGGATAACAGGGTAATCGATTT
	Reverse	GGATCCCGTACACGTAGTTCATTTTGCACAGGAAGCGCTCGGCGTCGTTGAGTGTGGC CAGACGCCAGTGTTACAACCAATTAACC
UL56 L144A	Forward	CAGCGTCAGTCTGGCCACACTCAACGACATCGAGCGCTTCGCGTGCAAAATGAACTAC GTGTATAGGGATAACAGGGTAATCGATTT
	Reverse	CCTCCGGCGCCAGGATCCCGTACACGTAGTTCATTTTGCACGCGAAGCGCTCGATGTC GTTGAGCCAGTGTTACAACCAATTAACC
UL56 L134A L137A I140A L144A	Forward	GGAGCTCCAGTTTACGGACAACGTCCGTTTCAGCGTCAGTGCGGCCACAGCCAACGAC GCCGAGCGCTTCGCGTGCAAAATGAACTACGTGTATAGGGATAACAGGGTAATCGATTT
	Reverse	CCTCCGGCGCCAGGATCCCGTACACGTAGTTCATTTTGCACGCGAAGCGCTCGGCGTC GTTGGCTGTGGCCGCACTGACGCTGAAACGGACGTGCCAGTGTTACAACCAATTAACC
UL56 I140A L144A	Forward	CAACGTCCGTTTCAGCGTCAGTCTGGCCACACTCAACGACGCCGAGCGCTTCGCGTGC AAAATGAACTACGTGTATAGGGATAACAGGGTAATCGATTT
	Reverse	CCTCCGGCGCCAGGATCCCGTACACGTAGTTCATTTTGCACGCGAAGCGCTCGGCGTC GTTGAGTGTGGCCAGACGCCAGTGTTACAACCAATTAACC
UL56 L134A L144A	Forward	GGAGCTCCAGTTTACGGACAACGTCCGTTTCAGCGTCAGTGCGGCCACACTCAACGAC ATCGAGCGCTTCGCGTGCAAAATGAACTACGTGTATAGGGATAACAGGGTAATCGATTT
	Reverse	CCTCCGGCGCCAGGATCCCGTACACGTAGTTCATTTTGCACGCGAAGCGCTCGATGTC GTTGAGTGTGGCCGCACTGACGCTGAAACGGACGTGCCAGTGTTACAACCAATTAACC

Table S3: Sequences of *UL56*-primers used for mutagenesis-PCR into putative zinc-finger pattern.

Mutant BAC	Primer direction	Primer sequence
UL56 C191S	Forward	GCCGGTGGCGCGTCAGGAAGTGTACGTCGAAGGGACGACATCTGCCCAATG CTACGAGGAGCTTAGGGATAACAGGGTAATCGATTT
	Reverse	CCTGATTCGGGATGATGGTCAGCTCCTCGTAGCATTGGGCAGATGTCGTCCC TTCGACGTACAGCCAGTGTTACAACCAATTAACC
UL56 C194S	Forward	GCGTCAGGAAGTGTACGTCGAAGGGACGACATGCGCCCAATCTTACGAGGA GCTGACCATCATTAGGGATAACAGGGTAATCGATTT
	Reverse	GCGAGCGGCCCTGATTCGGGATGATGGTCAGCTCCTCGTAAGATTGGGCGC ATGTCGTCCCTTGCCAGTGTTACAACCAATTAACC
UL56 C217S	Forward	TCAGGGCCGCTCGCTGAACAAGCGGCTGCAGGGCTTGCTGTCTAACCATATA GCGGTCCACCGTAGGGATAACAGGGTAATCGATTT
	Reverse	CATCGGACTGGCTTGACGGACGGTGGACCGCTATATGGTTAGACAGCAAGCC CTGCAGCCGCTGCCAGTGTTACAACCAATTAACC
UL56 N203A	Forward	GACATGCGCCCAATGCTACGAGGAGCTGACCATCATCCCGGCTCAGGGCCG CTCGCTGAACAATAGGGATAACAGGGTAATCGATTT
	Reverse	ACAGCAAGCCCTGCAGCCGCTTGTTCAGCGAGCGGCCCTGAGCCGGGATGA TGGTCAGCTCCTGCCAGTGTTACAACCAATTAACC
UL56 Q204R	Forward	ATGCGCCCAATGCTACGAGGAGCTGACCATCATCCCGAATAGAGGCCGCTCG CTGAACAAGCGTAGGGATAACAGGGTAATCGATTT
	Reverse	TGCACAGCAAGCCCTGCAGCCGCTTGTTCAGCGAGCGGCCTCTATTCGGGAT GATGGTCAGCTGCCAGTGTTACAACCAATTAACC
UL56 G205A	Forward	CGCCCAATGCTACGAGGAGCTGACCATCATCCCGAATCAGGCCCGCTCGCT GAACAAGCGGCTTAGGGATAACAGGGTAATCGATTT
	Reverse	GGTTGCACAGCAAGCCCTGCAGCCGCTTGTTCAGCGAGCGGGCCTGATTCG GGATGATGGTCAGCCAGTGTTACAACCAATTAACC
UL56 H219A	Forward	CCGCTCGCTGAACAAGCGGCTGCAGGGCTTGCTGTGCAACGCTATAGCGGT CCACCGTCCGTCTAGGGATAACAGGGTAATCGATTT
	Reverse	TATTCACATCGGACTGGCTTGACGGACGGTGGACCGCTATAGCGTTGCACAG CAAGCCCTGCAGCCAGTGTTACAACCAATTAACC
UL56 H223A	Forward	CAAGCGGCTGCAGGGCTTGCTGTGCAACCATATAGCGGTCGCCCGTCCGTC AAGCCAGTCCGATAGGGATAACAGGGTAATCGATTT
	Reverse	CCACCGTCTGGATATTCACATCGGACTGGCTTGACGGACGGGCGACCGCTAT ATGGTTGCACAGCCAGTGTTACAACCAATTAACC
